# Electroacupuncture for the management of symptom clusters in cancer patients and survivors (EAST)

**DOI:** 10.1186/s12906-023-03926-9

**Published:** 2023-03-27

**Authors:** Lifang Xie, Ding Quan Ng, Matthew Heshmatipour, Munjal Acharya, Paul Coluzzi, Nerida Guerrero, Sanghoon Lee, Shaista Malik, Ritesh Parajuli, Craig Stark, Rongwen Tain, Keri Zabokrtsky, Lilibeth Torno, Alexandre Chan

**Affiliations:** 1 Susan Samueli Integrative Health Institute, University of California Irvine Health, Irvine, CA USA; 2 Department of Clinical Pharmacy Practice, School of Pharmacy and Pharmaceutical Sciences, University of California Irvine, Irvine, CA USA; 3 School of Medicine, University of California Irvine, Irvine, CA USA; 4 Pacific Breast Cancer Center, University of California Irvine Health, Irvine, CA USA; 5 Department of Pediatric Hematology/Oncology, Hyundai Cancer Institute at Children’s Healthcare of Orange County, Orange, CA USA; 6 College of Korean Medicine, Kyung Hee University, Seoul, South Korea; 7 UCI Chao Family Comprehensive Cancer Center, Orange, CA USA; 8 The Facility for Imaging and Brain Research (FIBRE), University of California Irvine, Irvine, CA USA

**Keywords:** Electroacupuncture, Cancer, Symptom cluster, Integrative oncology, Randomized controlled trial

## Abstract

**Background:**

Neuropsychiatric symptoms, comprising cognitive impairment, fatigue, insomnia, depression, and anxiety, are prevalent and may co-occur during and after chemotherapy treatment for cancer. Electroacupuncture (EA), which involves mild electrical stimulation with acupuncture, holds great potential in addressing the management of individual symptoms. However, there is a lack of studies evaluating if EA can manage concurrent neuropsychiatric symptoms in cancer (i.e., symptom cluster). Hence, we designed a trial to evaluate the efficacy, safety, and feasibility of administering EA as an intervention to mitigate neuropsychiatric symptom clusters amongst cancer patients and survivors.

**Methods:**

The EAST study is a randomized, sham-controlled, patient- and assessor-blinded clinical trial. Sixty-four cancer patients and survivors with complaints of one or more neuropsychiatric symptom(s) in the seven days prior to enrollment are recruited from the University of California Irvine (UCI) and Children’s Hospital of Orange County (CHOC). Individuals with needle phobia, metastases, bleeding disorders, electronic implants, epilepsy, exposure to acupuncture in the three months prior to enrollment, and who are breastfeeding, pregnant, or planning to get pregnant during the duration of the study will be excluded. Screening for metal fragments and claustrophobia are performed prior to the optional neuroimaging procedures. Recruited patients will be randomized (1:1) in random blocks of four or six to receive either ten weekly verum EA (treatment arm, vEA) or weekly sham EA (control arm, sEA) treatment visits with a follow-up appointment four to twelve weeks after their last treatment visit. The treatment arm will receive EA at 13 acupuncture points (acupoints) chosen for their therapeutic effects, while the control arm receives minimal EA at 7 non-disease-related acupoints. Questionnaires and cognitive assessments are administered, and blood drawn to assess changes in symptom clusters and biomarkers, respectively.

**Conclusion:**

The EAST study can provide insight into the efficacy of EA, an integrative medicine modality, in the management of cancer symptom clusters in routine clinical practice.

**Trial registration:**

*This trial is registered with clinicaltrials.gov NCT05283577*.

## Background

As of January 2019, it was estimated that there were 16.9 million cancer survivors in the United States, and this number was projected to increase by 31.4%, to 22.2 million by 2030 and to 26.1 million by 2040 [1]. Many cancer survivors often experience a range of physical symptoms and neuropsychiatric burdens throughout and after their chemotherapy treatment, including fatigue, pain, insomnia, anxiety, alteration in cognitive function, and depression, reducing both quality of life and functionality [2]. It is estimated that up to 70% of cancer survivors experience six or more co-occurring symptoms [3]. Although pharmacologic and psychological treatments are used to treat specific symptoms, management of symptom clusters, defined as co-occurring related symptoms, remains challenging because none of these current pharmacological and psychological treatments are designed to address a broad range of symptoms that patients are experiencing concurrently [4]. Nonpharmacologic, complementary, and alternative medicine therapies, such as acupuncture, hold promise as a clinical treatment for the reduction of multiple co-occurring symptoms among cancer survivors.

Acupuncture is an integrative medicine technique involving the insertion of single-use, sterile fine needles into specific acupuncture points (acupoints) on the body linked to unwanted symptoms [5]. Acupuncture, which originated in Eastern Asia, has been employed widely to treat numerous disease conditions for over 2,500 years; its benefits have been recognized gradually by Western society [6]. Electroacupuncture (EA) was developed around the mid-1900s and is a modified form of acupuncture with a mild electric current passing between pairs of acupuncture needles. Although it is still not widely utilized, EA has been shown to achieve similar, or even better effects, compared to classical manual acupuncture [7]. Whereas the efficacy of acupuncture depends mainly on the manipulation technique of the individual acupuncturist, EA can be more reproducible by setting objective and quantifiable frequencies and intensities, thus making it more suitable for both basic and clinical research [7]. Recently, a study on integrative oncology service showed that acupuncture is one of the most frequently applied integrative oncology services in the United States and the European Union [8]. Evidence supports acupuncture as an effective clinical treatment for reducing individual symptoms of cancer-related fatigue [9–18], insomnia, [19–21] depression and anxiety [12, 14, 15, 22, 23], and cognitive impairment [24, 25]. This would be the first randomized controlled trial assessing the effects of acupuncture across symptom clusters in cancer survivors.

In this study, we aim to compare the efficacy of verum EA (vEA) treatment versus sham EA (sEA) in reducing cognitive toxicity, fatigue, psychological distress, insomnia, and to improve quality of life; to evaluate the impact of vEA versus sEA on biomarkers, including circulating brain-derived neurotrophic factor (BDNF), pro-inflammatory cytokines (IL-1β, IL-4, IL-6, IL-8, IL-10, TNF-alpha), mitochondrial DNA (oxidative stress indicator); to determine, in a sub-set of participants, the impact of vEA vs. sEA treatments on brain structure and function; and to assess the safety and feasibility of administering vEA to manage symptom clusters in cancer patients and cancer survivors for a future multicentered, pragmatic clinical trial.

## Methods

### Study design

This study is a randomized, sham-controlled, patient and assessor-blinded trial. The study protocol was approved by the institutional review board at the University of California, Irvine (UCI) and via reliance agreement with Children’s Hospital of Orange County (CHOC) and their institution’s respective institution review board (IRB). The protocol has been registered at ClinicalTrials.gov (NCT05283577). A total of 64 patients will be randomly assigned to a vEA or a sEA group in a 1:1 ratio. Forty of the 64 recruited participants will be recruited for the optional neuroimaging sub-study.

### Study locations

The administration of EA will occur at the Susan Samueli Integrative Health Clinic at the UCI main campus. The MRI for the optional neuroimaging sub-study will be done at the FIBRE imaging center, located at the UCI main campus, where we will also consent patients for our study, when applicable. The study will be utilizing the services of the Center for Clinical Research (CCR) at the UCI main campus for blood draws and administering the study questionnaires and the Cambridge Neuropsychological Test Automated Battery (CANTAB®) tests. Consenting of participants to the study will also occur at the CCR, when applicable.

### Participants

Participants will be recruited from the UCI Medical Centers by advertisements, practitioner referrals, and the UC Health Data Warehouse’s Honest Brokers System. We will also utilize the Consent-2-Contact Registry from the UCI Institute for Memory Impairments and Neurological Disorders (UCI MIND) for patients who previously expressed interest in clinical trials and who fit the eligibility criteria of the study [26]. Adolescent and young adult patients will be recruited from the Hyundai Cancer Institute at CHOC.

The study will be fully explained by a member of the study team and written informed consent will be obtained by a study investigator from each participant before entering the trial.

#### Inclusion criteria

1. Diagnosed with cancer and have received anti-cancer treatment.
2. ≥ 16 years of age.
3. Life expectancy ≥ 6 months.
4. Complaints of two or more of the following symptoms: Memory impairment/attention deficit, fatigue, insomnia, depression, or anxiety over the past seven days.
5. Able to provide informed consent to participate in the study.

#### Exclusion criteria

1. Cancer that has spread to one or more organs.
2. Severe needle phobia.
3. Severe psychiatric or medical disorders that would affect cognitive assessments.
4. Known bleeding disorder (e.g., hemophilia, Von Willebrand’s disease, thrombocytopenia).
5. Pacemaker or other electronic metal implants.
6. Epilepsy.
7. Received acupuncture therapy within the past three months prior to study enrolment.
8. Pregnant, breastfeeding, or are planning to get pregnant during the study period.

#### Exclusion requirements for optional functional MRI scan

1. Severe claustrophobia.
2. Non-removable metal orthodontic braces, metallic retainers, and oral wires.
3. Metal fragments in the body.

### Randomization, allocation, and blinding

After signing the informed consent, a total of 64 eligible participants will be stratified by recruitment sites [UCI (n = 34) or CHOC (n = 30)] and randomized in random blocks of four or six to receive either weekly vEA or weekly sEA. The practitioner who will perform the acupuncture treatment will then provide that intervention to the participant. The participants, outcome assessors, and statisticians will be blinded to the treatment allocation. Acupuncturist blinding cannot be achieved due to the nature of the intervention.

### Study procedures and time points

**Fig. 1:**
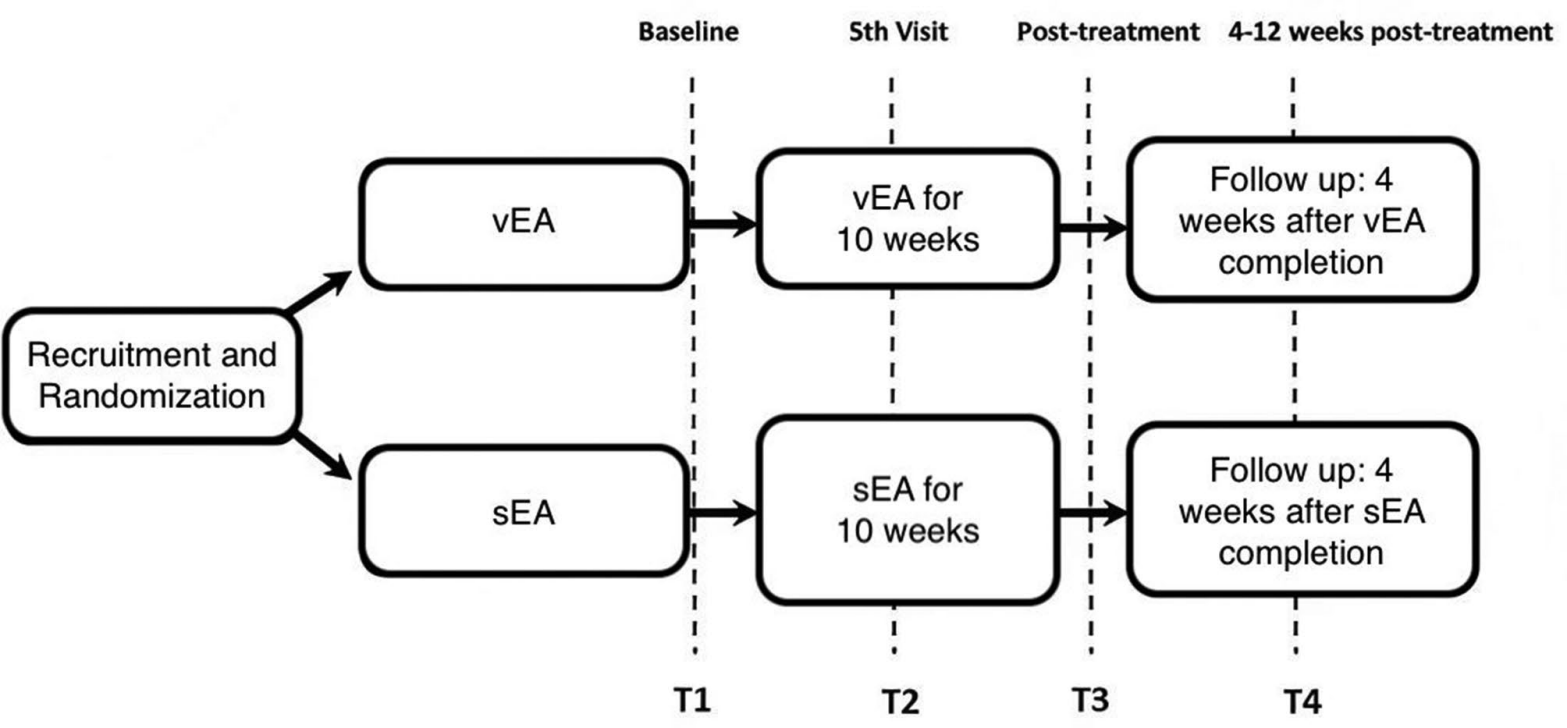
Study flow chart. Time point 1 (T1): Before initiation of verum EA or sham EA treatment; Time point 2 (T2): Between the 4th and 5th treatment visits; Time point 3 (T3): Within 1 week after the last planned verum EA or sham EA treatment visit; Time point 4 (T4): At least 4 weeks, and up to 12 weeks, after the last planned verum EA or sham EA treatment visit

Once a week for 10–12 weeks, participants will receive their respective treatments. The two-week buffer will be considered in the event of unexpected, missed treatment visits, such as illness or COVID-19 exposure. During the treatment period, there will be three time points at which participants will complete five questionnaires, a computerized neuropsychological test and have their blood collected for biomarker assessment. For the five questionnaires, study participants will be given the option to have the questionnaires administered before their treatment visit remotely. There will be a follow-up visit four to twelve weeks after their last EA treatment, where participants will again be assessed using the same questionnaires and cognitive tests and having their blood drawn for biomarker assessment. In terms of the optional neuroimaging aspect of our study, participants opting in will undergo an fMRI at baseline before they begin the treatments and after they finish their tenth and final treatment. In summary, the time points are as follows (Fig. 1):

- Time point 1 (T1): Before initiation of verum EA or sham EA treatment.
- Time point 2 (T2): Between the 4th and 5th treatment visits.
- Time point 3 (T3): Within 1 week after the last planned verum EA or sham EA treatment visit.
- Time point 4 (T4): At least 4 weeks and up to 12 weeks after the last planned verum EA or sham EA treatment visit.

### Study instruments

- AcuBEST brand disposable, sterilized stainless-steel acupuncture needles (size 0.20 × 25 mm) with FDA 510k approval for administering acupuncture treatment.
- Study data will be collected and managed using REDCap electronic data capture tools hosted at UCI [27, 28]. REDCap (Research Electronic Data Capture) is a secure, web-based software platform designed to support data capture for research studies, providing (1) an intuitive interface for validated data capture; (2) audit trails for tracking data manipulation and export procedures; (3) automated export procedures for seamless data downloads to common statistical packages; and (4) procedures for data integration and interoperability with external sources.
- OnCore™ clinical trial management system for storing patient information and assigning patient identifiers.
- EPIC Hyperspace at UCI and Cerner PowerChart at CHOC for accessing patient histories and other relevant information.
- CANTAB^®^ for assessing objective cognitive function.
- 3T Siemens Prisma scanner for fMRI neuroimaging.
- ELISA kits for measuring the concentration of biomolecules (e.g., IL-6) in the blood.
- Quantitative PCR (qPCR) machine for measuring mitochondrial DNA levels.

### Interventions

#### Verum EA group

Participants in the vEA group will receive acupuncture at disease-related acupoints: Shenting (GV24), Baihui (GV20), Sishencong (EX-HN1), Zhongwan (CV12), Guanyuan (CV4), Neiguan (PC6) bilateral, Shenmen (HT7) bilateral, Zusanli (ST36) bilateral, Sanyinjiao (SP6) bilateral, Taixi (KI3) bilateral, Zhaohai (KI6) bilateral, Hegu (LI4) bilateral, and Taichong (LR3) bilateral. With a supine position, needles will be subgaleally inserted at an angle of 15–30° along the scalp at GV24, EX-HN1, and GV20, while other body points listed above will be punctured perpendicularly. The depth of insertion will be 9–24 mm, depending on the location of the needle [29, 30]. Electrodes from the EA apparatus will be attached to each needle handle following needle insertion, and the EA stimulation will last for 30 min with a continuous wave of 2 Hz. The Baihui (GV20) and Shenting (GV24) needles are connected with electrodes. The four Sishencong (EX-HN1) electrodes are connected in pairs with one another. The Hegu (LI4) -Taichong (LR3), Neiguan (PC6)-Shenmen (HT7), Zusanli (ST36)-Sanyinjiao (SP6), and Taixi (KI3)-Zhaohai (KI6) pairs connected bilaterally. Finally Zhongwan (CV12) and Guanyuan (CV4) points are connected with one another. The EA intensity will be adjusted to be neither too strong nor uncomfortable depending on the individual’s threshold and sensitivity. Participants will receive one treatment session per week for 10 consecutive weeks for a total of 10 sessions. Locations of the acupoints are summarized in Table 1 [30, 31].

**Table 1:** Summary of the acupoint locations

Verum EA Acupoints	Location
Shenting (GV24)	On the head, 0.5 B-cun^a^ superior to the anterior hairline, on the anterior median line. When the anterior hairline is unclear or changed, GV 24 is located 3.5 B-cun superior to the midpoint between the medial ends of the eyebrows.
Baihui (GV20)	On the head, 5 B-cun superior to the anterior hairline on the anterior median line.
Sishencong (EX-HN1)	A group of four points at the vertex, 1 B-cun from GV20 in a cross-formation
Zhongwan (CV12)	On the upper abdomen, 4 B-cun superior to the center of the umbilicus, on the anterior median line.
Guanyuan (CV4)	On the lower abdomen, 3 B-cun inferior to the center of the umbilicus, on the anterior median line.
Neiguan (PC6)	On the anterior aspect of the forearm, between the tendons of the palmaris longus and the flexor carpi radialis, 2 B-cun proximal to the palmar wrist crease
Shenmen (HT7)	On the anteromedial aspect of the wrist, radial to the flexor carpi ulnaris tendon, on the palmar wrist crease.
Hegu (LI4)	On the dorsum of the hand, radial to the midpoint of the second metacarpal bone
Zusanli (ST36)	On the anterior aspect of the leg, about one finger-breadth lateral to the tibia, 3 B-cun inferior to ST35 (On the anterior aspect of the knee, in the depression lateral to the patellar ligament).
Sanyinjiao (SP6)	On the tibial aspect of the leg, posterior to the medial border of the tibia, 3 B-cun superior to the prominence of the medial malleolus.
Taixi (KI3)	On the posteromedial aspect of the ankle, in the depression between the prominence of the medial malleolus and the calcaneal tendon.
Zhaohai (KI6)	On the medial aspect of the foot, 1 B-cun inferior to the prominence of the medial malleolus, in the depression inferior to the medial malleolus.
Taichong (LR3)	On the dorsum of the foot, between the first and second metatarsal bones, in the depression distal to the junction of the bases of the two bones, over the dorsalis pedis artery.

^a^This method uses landmarks on the body surface, such as joints, divides the length between two points into equal portions and locates acupuncture points by such proportions. Each portion equals 1 B-cun.

#### Sham EA group

Participants in the sEA group will receive acupuncture at non-disease related acupoints: Pianli (LI6) bilateral, Wenliu (LI7) bilateral, Fuyang (BL59) bilateral, Kunlun (BL60) bilateral, Sanyangluo (TE8) bilateral, Sidu (TE9) bilateral, and Daheng (SP15) bilateral with a superficial insertion and 2 Hz stimulation on each acupoint continuously for 30 min. The sEA provides minimal electrical stimulation with no distinguishable difference in sound as vEA.

Throughout the trial, all the participants will be treated separately to prevent communication. Furthermore, to ensure proper reporting of our acupuncture-based study, we referenced the STRICTA 2010 checklist shown in Table 2 [32].

**Table 2:** STRICTA 2010 checklist

Item	Detail
**1. Acupuncture rationale**	1a) Style of acupuncture - **Traditional Chinese Medicine**
	1b) Reasoning for treatment provided, based on historical context, literature sources, and/or consensus methods, with references where appropriate – **Expert group consensus based on historical context**
	1c) Extent to which treatment was varied - No variation
**2. Details of needling**	2a) Number of needle insertions per subject per session (mean and range where relevant) – **vEA (13 acupoints, total 24 needles including bilateral ones) vs. sEA (7 acupoints, total 14 needles including bilateral ones)**
	2b) Names (or location if no standard name) of points used (uni/bilateral) - **See** Table 1.
	2c) Depth of insertion, based on a specified unit of measurement, or on a particular tissue level **vEA 9–24 mm, 15–30° at GV24, EX-HN1, and GV20, and 90° at other body points. 2 Hz individually adjusted EA intensity.** **sEA - a superficial insertion, 2 Hz minimal EA stimulation with the same EA sounds**
	2d) Response sought (e.g. *de qi* or muscle twitch response) - **vEA with de qi, sEA without de qi**,
	2e) Needle stimulation (e.g. manual, electrical)- **EA**
	2f) Needle retention time- **30 min**
	2 g) Needle type (diameter, length, and manufacturer or material) - **size 0.20 × 25 mm, AcuBEST(South El Monte, USA)**
**3. Treatment regimen**	3a) Number of treatment sessions − **10 sessions**
	3b) Frequency and duration of treatment sessions − 1 per week * 30 min
**4. Other components of treatment**	4a) Details of other interventions administered to the acupuncture group (e.g. moxibustion, cupping, herbs, exercises, lifestyle advice)- **N/A**
	4b) Setting and context of treatment, including instructions to practitioners, and information and explanations to patients - **a randomized, sham-controlled, patient and assessor-blinded trial**
**5. Practitioner background**	5) Description of participating acupuncturists (qualification or professional affiliation, years in acupuncture practice, other relevant experience) - T**wo California licensed acupuncturists with PhD degrees with more than 20 years of acupuncture practice.**
**6. Control or comparator interventions**	6a) Rationale for the control or comparator in the context of the research question, with sources that justify this choice - **non-disease related acupoints**
	6b) Precise description of the control or comparator. If sham acupuncture or any other type of acupuncture-like control is used, provide details as for Items 1 to 3 above - **Mentioned in 1 to 3**

### Sample size calculation

Based on our previous psychometric study of the Functional Assessment of Cancer Therapy – Cognitive Function (FACT-Cog) questionnaire, a decrease of 6.9–10.6 points (4.7–7.2% of the total score) in the FACT-Cog corresponds to the threshold for clinically significant cognitive deterioration in breast cancer patients [33]. In our previous prospective cohort study, among 131 participants who completed the study, their mean and standard deviation (SD) of FACT-Cog total score prior to chemotherapy initiation, six weeks following chemotherapy initiation (end of cycle 2), 12 weeks following chemotherapy initiation (end of cycle 4), and approximately 15-months post-chemotherapy initiation (post‐chemotherapy evaluation) were estimated to be 132.00 (SD 15.65), 130.23 (19.44), 128.51 (19.93) and 127.53 (21.89), respectively [34]. Assuming the correlation between observations on the same participant across time is 0.2 and a common standard deviation of two groups is 20.5, with 29 evaluable participants per group, a total of 58 evaluable participants, a power of 80% will be achieved to detect the difference of 9.6 in means of FACT-Cog total score between two groups across four time points with a p value of 0.05. After accounting a potential 10% attrition, 64 eligible patients will be enrolled into the study. Forty of the 64 participants will be recruited for the optional fMRI neuroimaging study.

### Outcomes

#### Primary outcome measures

Self-perceived cognitive function **–** All study participants will complete the FACT-Cog version 3 questionnaire to assess self-perceived subjective cognitive function.

#### Secondary Outcome Measures

- Objective cognitive function **–** All study participants will complete CANTAB^®^, to assess objective cognitive functions. CANTAB^®^ is a computerized cognitive testing software to assess selected cognitive domains of memory (paired associates learning, PAL), response speed (reaction time, RTI), executive function (spatial working memory, SWM; multitasking test, MTT) and attention (rapid visual information processing, RVP). Both self-perceived and objective assessments are recommended by the International Cognition and Cancer Task Force (ICCTF) [35].
- Fatigue – Multidimensional Fatigue Symptom Inventory – Short Form (MFSI-SF) is a validated questionnaire comprises of 30 items and contains five subscales (general fatigue, physical fatigue, emotional fatigue, mental fatigue, and vigor), each with six items.
- Psychological distress and insomnia – The Rotterdam Symptom Checklist (RSCL) will be used to measure psychological symptoms (anxiety and depression) and insomnia. Psychological distress is indicated by a score of greater than 16 in the psychological domain. Insomnia is measured by a single item in the checklist.
- Quality of life
  The European Organisation for Research and Treatment (EORTC) Quality of Life of Cancer Patients (QLQ-C30) is a validated questionnaire for assessing the health-related quality of life of cancer patients participating in international clinical trials. It incorporates five functional scales (cognitive, emotional, physical, role, and social), several symptom scales (e.g., pain, fatigue, insomnia), and a global health scale.
  The EuroQol Research Foundation EQ-5D-5 L comprises a visual analog scale of general health status and a descriptive system based on five dimensions of health status: mobility, self-care, usual activities, pain/discomfort, and anxiety/depression.
- Plasma biomarker assessment – 10 mL of peripheral blood will be collected from each study participant in purple (or lavender) top K2 EDTA tubes at four timepoints: Baseline, before initiation of treatment with vEA or sEA; between fourth and fifth treatments; within one week of completing treatment; and four to 12 weeks after completing treatment. The EDTA tube will be centrifuged at 1000 × g for 10 min within 30 min of collection. Plasma will be aliquoted and stored aseptically at -80 °C until analysis. Plasma BDNF and cytokines (IL-1β, IL-4, IL-6, IL-8, IL-10, TNF-alpha) levels will be quantified using commercially available ELISA kits. Mitochondrial DNA will be quantified using quantitative PCR. Please refer to our previous studies for analytical methods [36–38].
- Neuroimaging **–** In a subset of study participants recruited from UCI, gray matter and white matter volumes, diffusional weighted imaging (DWI) measures (mean diffusivity, fractional anisotropy, radial, and axial diffusivities as well as higher-order NODDI metrics), and resting state functional connectivity of different neural networks will be assessed using MRI at 3T using a Siemens Prisma scanner.
- ﻿Feasibility
  *Recruitment*: Recruitment will be evaluated as the number of participants recruited (% of target recruitment) and rate of recruitment a month. Reasons for declining the participation will also be documented. Additionally, time spent on recruitment will be examined to assess recruitment productivity.
  *Compliance*: Compliance will be measured as the number of acupuncture sessions successfully completed, and the proportion of participants completing the scheduled acupuncture sessions.
  *Blinding and patient acceptance*: All participants will complete a questionnaire created by the investigators evaluating their perceptions towards the EA treatment at the end of study period. Participants will be asked whether they believe that they have received vEA or sEA, if they are satisfied and benefited from the treatment, and whether they would consider undergoing treatment again outside of a trial setting.
- Safety – Participants will be monitored for adverse events such as feeling faint after acupuncture treatment, minor bruising, pain or discomfort, bleeding, and possible infections. Severity will be graded, when appropriate, according to the Common Terminology Criteria for Adverse Events (CTCAE) version 5. All adverse events will be reported immediately and recorded in the case report form (CRF).

### Statistical analyses

Categorical variables (participants recruited, acupuncture sessions completed, participants completing all sessions, adverse events, participant responses to acceptability questionnaire) will be analyzed using descriptive statistics, presented as counts and percentages. All the descriptive statistics will be constructed for the entire cohort and stratified by the treatment allocation. All the mean scores of symptoms and quality of life measures gathered from the different questionnaires will be compared at T1 (baseline), T2, T3, and T4 for vEA and sEA groups. The mean score changes will also be compared between the vEA and sEA groups at T2, T3, and T4. Biomarkers (BDNF, cytokines, and mitochondrial DNA level) changes will be compared between treatment groups. Mixed effects model, with random intercepts for individual participants, will be generated to evaluate the changes of symptoms and biomarkers over time, and its association with the treatment allocation (vEA or sEA). The covariates of interest include the treatment indicator (vEA or sEA), the time variable, and the interaction term treatment x time. Both intention-to-treat and per-protocol analyses will be conducted. A significance level of 0.05 will be used, and all statistical analyses will be performed using Stata 16.

### Data collection and management

To warrant quality of the data, a well-trained assessor will be responsible for data collection and recording on the CRF on Microsoft Teams. Double entry of the data into REDCap will be implemented by clinical research coordinators. Regular monitoring of the recruitment, intervention, and assessment processes will be performed to ensure predetermined protocol and standard operating procedures are followed. All study documentation will be stored in a locked cabinet in an area with limited access. In addition, participants will be registered on the electronic health record (EHR) system, EPIC, where they will have their information secured electronically. Participant information will also be stored in OnCore, a secure clinical trial management system.

### Study dissemination

In addition to disseminating our results to ClinicalTrials.gov and peer-reviewed journals, we intend to present results at relevant conventions and professional meetings.

## Discussion

Patients receiving chemotherapy face a group of unpleasant physical and psychological symptoms, such as fatigue, anxiety, depression, sleep disturbance, and cognitive impairment, often co-occurring as symptom clusters. These symptom clusters can have profoundly negative effects on patients’ quality of life.

Numerous studies have indicated that oriental medicine not only alleviates symptoms (e.g., fatigue, chronic pain, anorexia/cachexia, and insomnia) of patients with cancer and improves their quality of life but also diminishes adverse reactions and complications caused by chemotherapy or radiotherapy. Acupuncture, one of the major components of oriental medicine, is an available complementary therapy to relieve cancer- and treatment-related complications. More importantly, EA is readily replicable due to its standardized interventional approach [7].

This study will be the first clinical trial that assesses the effects of EA across multiple symptom clusters in cancer patients and survivors. The findings from this trial will be an encouraging step toward expanding survivorship care to meet the needs of the growing cancer survivor population. Understanding symptom clusters in cancer survivors may result in greater therapeutic benefits by integrating treatments for concurrent symptoms, thus improving quality of life [39]. In addition to quality of life, mitigation or complete resolution of multiple symptoms will also positively affect prognosis and functional status of those afflicted [40]. The hypothesis from this study, to target comorbid cognitive, affective, and somatic symptoms simultaneously, will open new avenues of exploration. This study will also uniquely leverage a translational approach to understand how objective biomarkers are linked with the change of various symptoms over treatment. Results of this trial will propel the use of evidence-based integrative health treatment modalities in managing cancer patients’ well-being.

## Conclusion

Overall, this study will determine the efficacy of utilizing EA as an intervention for treating symptom clusters in cancer patients and survivors. If successful, the results of our study can provide clinical significance in implementing integrative medicine as a modality in the management of symptom clusters in cancer patients and survivors.

## Data Availability

The data collected and presented for our research can be found at ClinicalTrials.gov **NCT05283577**.

## Declarations

### Trial status

Our trial has been approved by the UCI IRB (IRB #20216732). We foresee the study will be completed in the first quarter of 2024.

## Abbreviations

EA: Electroacupuncture
B-cun: Proportional Bone (skeletal) cun
vEA: Verum Electroacupuncture
sEA: Sham Electroacupuncture
RCT: Randomized Controlled Trial
